# Association between irrigation thresholds and promotion of soil organic carbon decomposition in sandy soil

**DOI:** 10.1038/s41598-021-86106-4

**Published:** 2021-03-24

**Authors:** Jean-Pascal Matteau, Paul Célicourt, Guillaume Létourneau, Thiago Gumiere, Christian Walter, Silvio J. Gumiere

**Affiliations:** 1 Department of Soil and Agricultural Engineering, Laval University, Quebec City, QC Canada; 2 Institut Agro-INRA, UMR 1069, Sol Agro et hydrosystéme Spatialisation, 35000 Rennes, France

**Keywords:** Environmental sciences, Hydrology

## Abstract

Soil organic carbon (SOC) has a significant effect on the carbon cycle, playing a vital role in environmental services and crop production. Increasing SOC stock is identified as an effective way to improve carbon dioxide sequestration, soil health, and plant productivity. Knowing soil water is one of the primary SOC decomposition driver, periods in the crops growth stages with increased water movement might influence the SOC dynamics. Here, we evaluate the temporal effect of four precision irrigation thresholds ($-15$, $-30$, $-45$, and $-60$ kPa) in potato crop on SOC dynamics using the Partial Least Square algorithm and the Tea Bag Index in a sandy soil under potato production. The difference of SOC decomposition rate between the precision irrigation thresholds is developed in the second quarter of the growing season, between 38 and 53 days after planting. This critical period occurred in a stage of strong vegetative growth and rapid irrigation cycles. The precision irrigation threshold affected the decomposition rate of SOC. A faster decomposition of labile organic carbon was promoted by water excess ($-15$ kPa). The dryer ($-30$, $-45$, and $-60$ kPa) precision irrigation thresholds did not show any differences. The advancement of this knowledge may promote soil health conservation and carbon sequestration in agricultural soil.

## Introduction

Soil organic matter (SOM) contains more than three times as much carbon as the atmosphere or the terrestrial vegetation^1^. As part of SOM, the SOC has a significant effect on the carbon cycle, acting as a carbon sink or source^2^. Furthermore, SOC plays a vital role in environmental services and crop production^3^. SOC stocks can increase crop yield and soil health by increasing available water capacity, improving the supply of nutrients, and enhancing soil structure and other physical properties^4^. SOC was also identified as one of the dominant factors in assessing soil quality^5^. Thus, increasing SOC stock is identified as an effective way to improve carbon dioxide sequestration, soil health, and plant productivity.

Optimal water management and precision irrigation techniques, ensuring the crops an optimal amount of water at the right time to sustain all physiological process, help increase plant productivity and water use efficiency in a variety of cultures, including celery, onion^6^, tomato^7,8^, lettuce^9^, strawberry^10^, cranberry^11,12^, and potato^13,14^. In this regard, soil matric potential (SMP) has proven to be a reliable criterion to characterize soil water availability^15^. This sensing technique provides an approximation of the actual crop water requirement by emphasizing the soil-water interaction, strongly linked with the soil-plant interaction and the plant water requirement. It is well known that soil moisture impacts SOC dynamics, acting as one of its three primary drivers alongside temperature and soil biodiversity^16–19^. Therefore, we hypothesize that periods in the crops growth stages with increased water movement might influence the SOC dynamics. Few information is available on the temporal effect of soil moisture levels (i.e., precision irrigation thresholds) on the SOC dynamics. However, such an effect can be monitored using a precision irrigation system and a soil sensor network and analyzed using a partial least square (PLS) method^20^. PLS is an efficient machine learning technique suitable to model complicated relationships between predictors and responses. It performs well not only when the predictor variables are large, but also when there exists strong collinearity between them^21–23^, which is the case in studies of environmental factors, like soil matric potential (SMP) effects on SOC dynamics^23–27^. A comparative analysis has been made, identifying the PLS as more reliable than Principal Components Analysis (PCA) and Multiple Regression (MR) or a combination of both to identify the magnitude of influence of relevant variable^28^.

Traditional methods to assess the SOC dynamics, like dry combustion or litter bags, can be time and resources consuming^29,30^. However, the Tea Bag Index (TBI) method^30^ is a simple, cost–effective, and well–standardized approach to gather data on decomposition rate and litter stabilization using commercially available tea bags susceptible to be fed into a PLS algorithm. The TBI method has been previously used and found useful to study SOC decomposition and dynamics^30,31^. It is suitable to analyze the effects of interactions between plants, microorganisms, and mineral phases, as well as the evolution of labile and recalcitrant organic matter. This evolution is captured through two parameters: the decomposition rate (k) and the litter stabilization factor (S).

The purpose of this study was to evaluate the temporal effect of precision irrigation thresholds on SOC decomposition dynamics. In a greenhouse experiment, a soil matric potential sensor network was used in combination with the TBI method and a PLS algorithm to assess the temporal effect of precision irrigation thresholds on SOC dynamics. The hypothesis tested is that irrigation directly affects the rate of decomposition of organic matter in the soil under potato planting. To the authors’ knowledge, this is the first study that presents the water management effect on SOC distributed over repeated growing seasons using organic matter dynamics measurement and the PLS statistical method. Improving soil water management may optimize soil carbon sequestration, reducing greenhouse gas emissions in cropping systems.

## Results

### Identification of critical periods

The effect of precision irrigation thresholds on labile SOC decomposition rate (k) was distributed along the growing season using the PLS. The daily averages of soil matric potential were used as principal components in the PLS, and the number of days after planting (DAP) with an importance over 85 for the k determination were selected as critical periods.

**Figure 1:**
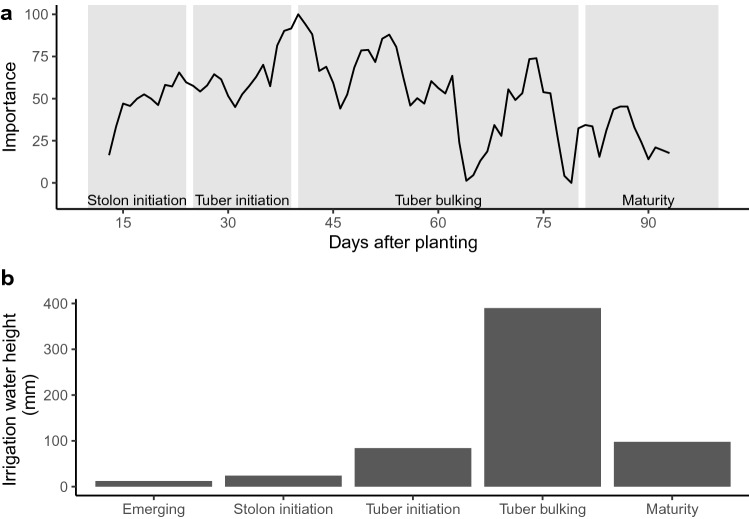
(**a**) Seasonal distribution of the PLS principal component importance in the prediction of k according to the potato’s physiological growth stage. (**b**) Mean irrigation water height according to the physiological growth stage of the potato for the $-15$ kPa irrigation threshold.

Critical periods in the determination of k are set between the 38 and 53 days after planting. As shown in Fig. 1a, the PLS principal components importance is maximized in the tuber initiation and early tuber bulking stage. The importance presented in the graph corresponds to the contribution of daily SMP values across the experimental unit to the SOC decomposition rate (k) values. The importance indicates that the critical period of the plant growth corresponds to periods where the daily SMPs across the units contribute the most to the development of the decomposition rate (k). As shown in Fig. 1b, maximal irrigation volume occurs in the tuber bulking stage, as expected. Surprisingly, over the three experiments, the tuber initiation stage required a small irrigation volume.

**Figure 2:**
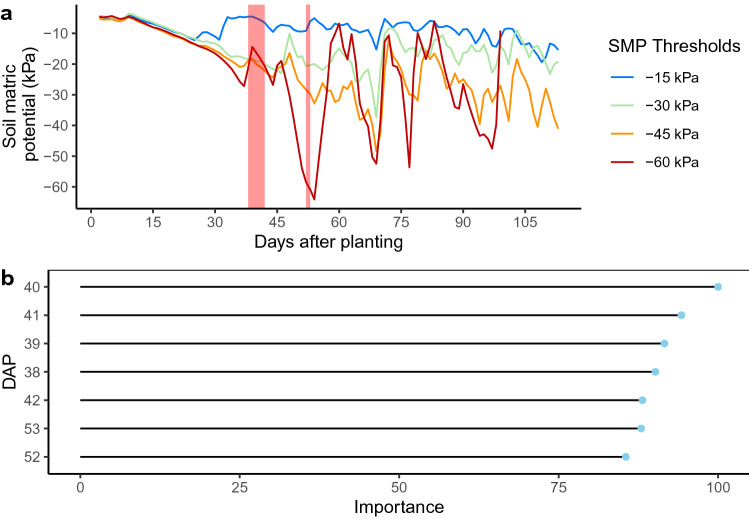
(**a**) Daily average soil matric potential (lines) of Winter 2018 with PLS identification of critical periods (red polygons). The importance reaches its peak between 38 and 42 days after planting (DAP), the first red polygon, and between 52 and 53 DAP. (**b**) Zoom of the days after planting with an importance of over 85.

The difference between the precision irrigation thresholds is made early in the season as most of the PLS principal components correspond to SMP in the second quarter of the growing season, as shown in Fig. 2a and is maintained until harvest. The critical periods identified occurred between 38 and 53 DAP, as shown in Fig. 2b. The critical periods identified occurred in the tuber initiation and early tuber bulking stage. These growing stages correspond to young plants with strong vegetative growth. Irrigation cycles and SMP are also subject to rapid and significant variation in the tuber initiation and early tuber bulking stage.

### Teabags decomposition rate and stabilization factor

**Figure 3:**
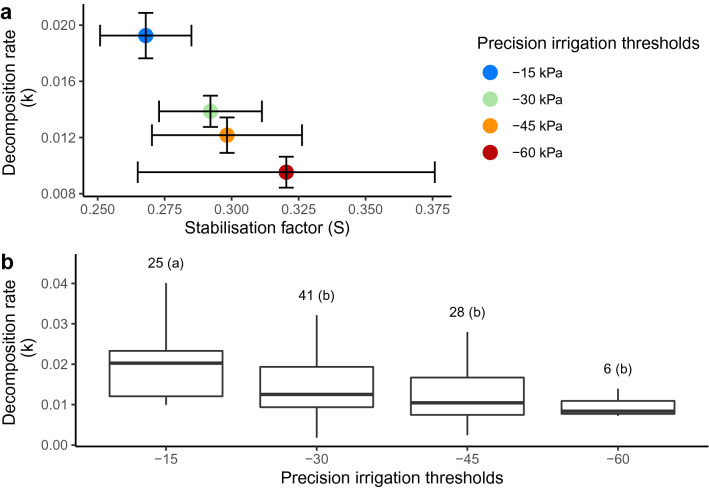
(**a**) The mean decomposition rate k (g g$^{-1}$ d$^{-1}$) and stabilization factor S of the tea bag index for each precision irrigation thresholds (colors) and experiment (shapes). Each point contains four replicates. Error bars are standard errors. (**b**) Decomposition rate (k) for each precision irrigation threshold. The numbers are the replicates of each treatment. The precision irrigation thresholds with different letters in parenthesis are significantly different.

Figure 3 shows the effect of precision irrigation thresholds on the Tea Bag Index parameters (k and S) and the total SOC. The $-15$ kPa precision irrigation threshold showed the strongest effect on the decomposition rate (k) and was significantly higher than all other thresholds (*p*-value = $0.000663$). The stabilization factor was not significantly affected by precision irrigation thresholds.

Figure 3 shows the distribution of k and S between precision irrigation thresholds and the experiment. The averaged decomposition rate (k) by precision irrigation thresholds ranged from 0.0095 g g$^{-1}$ d$^{-1}$ ± 0.0011 (SE) to 0.0193 g g$^{-1}$ d$^{-1}$ ± 0.0016 (SE). The averaged stabilization factor (S) by precision irrigation thresholds ranged from 0.268 ± 0.017 to 0.32 ± 0.055 (dimensionless).

### Weight loss-on-ignition seasonal variation

**Figure 4:**
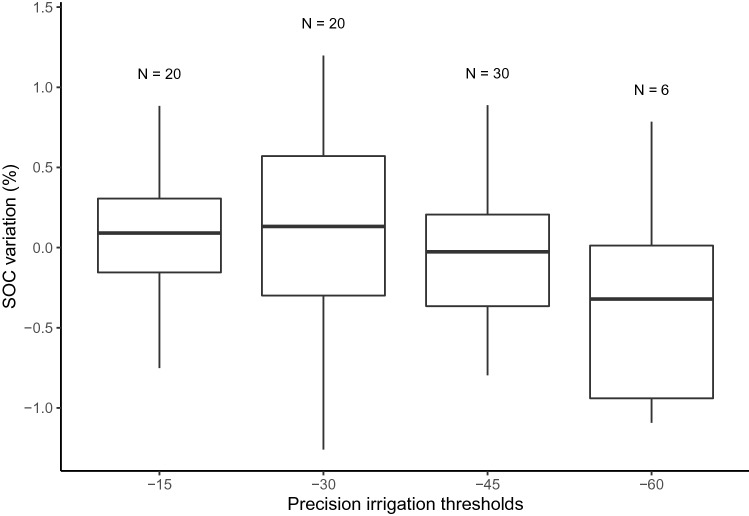
Variation of soil organic carbon between seeding and harvest for three greenhouse experiments.

Weight loss-on-ignition did not differ significantly between precision irrigation thresholds. Figure 4 shows that organic matter percent variation is either negative or positive with a median close to zero for the $-15$, $-30$, and $-45$ kPa thresholds, showing neither a general trend of SOC accumulation nor decomposition. The $-60$ kPa precision irrigation threshold lacks replicates to show effect in the statistical analysis.

## Discussion

In this study, the daily mean soil matric potential data were considered as a spectrum in the PLS to identify critical periods in the determination of the decomposition rate of labile organic carbon. Results suggest that the PLS component importance does not depend on irrigation volume or irrigation frequency. Results suggest that with a larger dataset, PLS used with soil matric potential as a spectrum could be used to predict SOC decomposition rate. The critical periods identified with the PLS are set between 38 and 53 days after planting, which corresponds to the potato physiological stage of tuber initiation and early tuber bulking. These are two critical physiological stages in potato production, especially for potato irrigation^32^. The root water uptake is growing in both stages, and it culminates in the tuber bulking stage with the highest water demand^33^. The results underline a connection between the SOC decomposition rate and the potato water uptake. They indicate that tuber initiation and tuber bulking are the most critical periods during which irrigation must be managed accurately to prevent fast labile organic carbon decomposition.

The results also indicate that soil water retention time and the frequency of wetting-drying cycles may influence SOC dynamics. In our study, the soil matric potential of $-15$ kPa represents an irrigation threshold that reduces water retention time while increasing the frequency of wetting-drying cycles. Researchers showed that frequent wetting-drying cycles might increase SOM mineralization^34^. Wetting-drying cycles negatively affect cumulative soil respiration (SOC release), which is exacerbated if the moist period after re-wetting is short^35^. Furthermore, for a fixed incubation period, the cumulative soil respiration increases with regular and sufficient soil moisture management and decreases over the drying periods before a re-wetting event^36^. Our study shows that the SOC decomposition rate decreases with decreasing soil matric potential thresholds and increasing periodicity in drying-wetting cycles.

The precision irrigation thresholds of $-15$ kPa significantly increased the SOC decomposition rate (k), suggesting a long-term precision irrigation thresholds of $-15$ kPa SMP could lead to a depletion of SOC stock in sandy soil. The results suggest that water management effect on SOC dynamics is shown on the short-term decomposition of labile carbon, as previously suggested^31,37^. Precision irrigation thresholds can also play a role in the direction of long-term carbon fixation as previously shown, a soil matric potential irrigation threshold of $-25$ kPa contained the most relative abundance of microorganisms predicted genes related to carbon fixation and emission; other precision irrigation thresholds ($-15$, $-30$ and $-45$ kPa) were significantly lower^38^. These results qualify the previous assessment that applying irrigation on sandy soil increases SOC^39^.

Soil matric potential has also been found a suitable method to assess the effect of water, and oxygen, on soil carbon dynamics. A difference of soil volumetric water content between 0.33 and 0.35 cm$^3$/cm$^3$ was sufficient to create a significant difference in k. These differences are equivalent to the difference between $-15$ and $-30$ kPa of soil matric potential for the soil used in this study. Therefore, the SOC decomposition rate (k) decreases when the soil volumetric water content decreases from 0.35 to 0.33 cm$^3$/cm$^3$. Indicating that even a minimal change in soil moisture from one threshold to another can significantly affect the SOC decomposition rate.

We have found that the Tea Bag Index method is suitable to assess the effects of interactions between plants and soil water on SOC dynamics. The TBI results obtained in our greenhouse system showed a decomposition rate in the usual range, as they ranged in the literature from 0.005 to 0.04^30,31,37^, and a high stabilization factor in the comparison of other irrigation experiments^31,37^. Previous studies showed that the Tea Bag Index results are consistent with those using native litters^40^. The tea leaves also showed a typical decomposition compared with other plant litters^41^, behaving like dead roots or straws.

The precision irrigation thresholds did not significantly affect the SOC variation measure using the weight loss-on-ignition method. This method measures the total soil organic carbon present in the soil. The SOC of the soil used in this study was 17.98 g kg$^{-1}$, which is slightly above the world average (13 g kg$^{-1}$) and far from the higher concentrations (195 g kg$^{-1}$)^39^. The low initial carbon concentration and the short experiment time are two factors known to limit the effect of irrigation on SOC variation^19^.

Surprisingly, over the three experiments, the tuber initiation stage required a small irrigation volume as 14 % of the water was used in the tuber initiation stage compared to 64 % in the tuber bulking stage. Water stress occurring in the tuber initiation stage can limit photosynthesis, total biomass, and yield^42^, limiting the number of tubers produced by each plant^43^. Thus, water stress occurring in the tuber bulking stage affects yield and tuber quality^44,45^. The PLS algorithm could also be a useful tool to determine the most critical stage in the potatoes water demand.

## Conclusion

In this study, the effect of precision irrigation threshold on SOC decomposition rate was evaluated using the tea bag index in potato crops. The results indicate that faster decomposition of labile organic carbon was promoted by water excess ($-15$ kPa) and increased irrigation frequency. The PLS algorithm was used to identify the crop growth stage periods that determine the decomposition rate using the daily SMP values from the experimental units as input variables. The effect on the decomposition rate (k) between the precision irrigation thresholds is developed in the second quarter of the growing season, between 38 and 53 days after planting. This critical period occurred in a stage of strong vegetative growth and rapid irrigation cycles. Also, PLS results suggest that with a larger database, PLS using daily soil matric potential could be used to predict SOC decomposition. Evidence provided in this study shows that irrigation is an important driver of the SOC decomposition rate. The tea bag index has been found more sensitive to decomposition in one growing season than the weight loss-on-ignition method. Future research should evaluate the effect of irrigation on SOC decomposition in different soil types and climates in field experiments. The linkage between SOC decomposition and SOC sequestration also needs some further investigation.

## Materials and methods

### Experimental design

**Figure 5:**
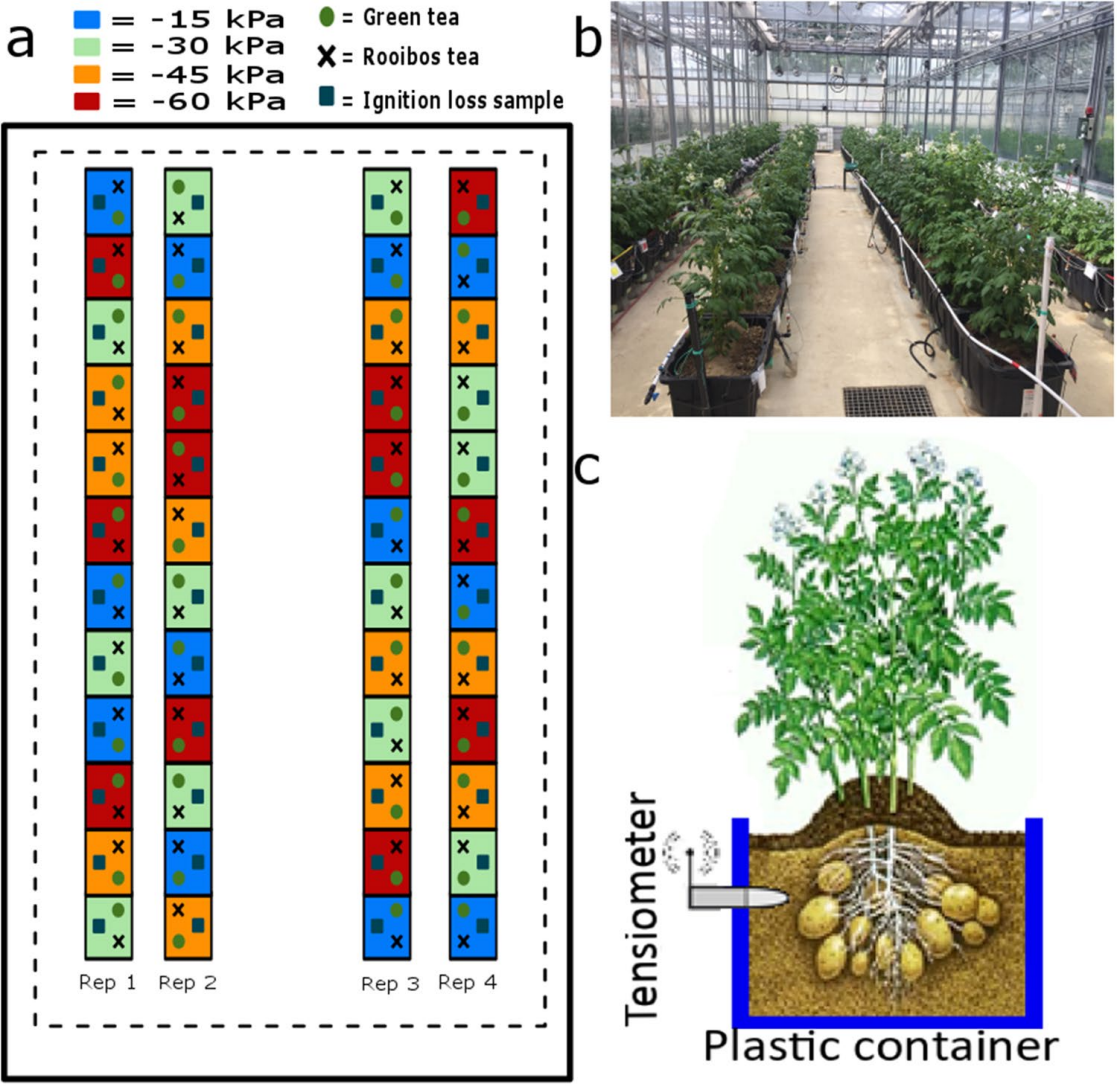
(**a**) Scheme of the experimental design in winter 2018 in the greenhouse, including four precision irrigation thresholds ($-15, -30, -45$, and $-60$ kPa), two tea types, and weight loss-on-ignition samples aggregated in 48 experimental units. (**b**) Picture of the greenhouse experiment. (**c**) Scheme of the short side of an experimental unit with one tensiometer.

We conducted three experiments in the high-performance greenhouse complex of Laval University (Québec, Qc, Canada) in 2018 (1 experiment) and 2019 (2 experiments). The experiments consisted of cultivating potato in 48 (2018) and 60 experimental units (2019) of 0.14 m$^3$ ($60 \times 40 \times 40$ cm) using four soil matric potential thresholds($-15$, $-30$, $-45$, and $-60$ kPa) for every 12 (2018) and 15 (2019) units (Fig. 5). We planted two tubers at a depth of 7.5 cm and installed one tensiometer at 10 cm depth in each experimental unit. Plants were provided as seeds by the cooperative ”LA PATATE LAC-SAINT-JEAN” (Quebec, Canada). The experiments conducted are in accordance with relevant institutional and national guidelines. Data from the tensiometer were collected at two minutes intervals and used to trigger irrigation automatically.

Individual irrigation lines were used to control irrigation independently for each precision irrigation threshold, allowing accurate irrigation management. Irrigation duration throughout the growing season was adjusted to reach a SMP between $-1$ and $-5$ kPa after irrigation. Irrigation water was applied using drip irrigation emitters placed on the soil surface. From potato seeding to emergence, irrigation was the same for all thresholds to ensure uniform emergence. The irrigation experiment began with the potato emergence and went on until harvest.

The soil temperature and water content were measured in 12 experimental units (three units per SMP threshold randomly selected) in 2018 at 10-cm depth using GS3 probes (Decagon Devices, Inc., Pullman, WA, USA) at 15 minutes intervals.

### Soil chemical and physical characteristics

The soil was sampled from a potato field at Dolbeau-Mistassini city ($48^\circ ~ 51^\prime ~ 31''$ N and $72^\circ ~ 11^\prime ~ 50''$ W). It was collected from the topsoil (0-20 cm), homogenized, and placed into the plastic container. The soil was a Podzolic sandy soil according to the Canadian soil classification^46^. Table 1 shows the soil properties and its van Genuchten parameters^47^. Particle size distribution was determined by the sedimentation method^48^. Fertilization was carried out following the local crop recommendation for potato^49^. The available nutrients concentration (P, K, Ca, Mg, and Al) was determined using the Mehlich III extraction method^50^ and determined at the Institut de Recherche et de Développement en Agroenvironnement (IRDA, Sainte-Foy, QC, Canada) using inductively coupled plasma optical emission spectrometry (ICP–OES) (Model ICAP; PerkinElmer, Boston, MA). The soil has been saturated with water before planting and was replaced between experiments to avoid soil-borne diseases.

**Table 1:** Soil properties and optimized van Genuchten parameters.

Clay (%)	Silt (%)	Sand (%)	Organic C (g kg$^{-1}$)	pH	$\theta _r$	$\theta _s$	$\alpha$ ($\hbox {cm}^{-1}$)	n
0	8.2	91.8	17.98	5.01	0.053	0.48	0.195	2.13

$\theta _r$ is the residual water content, $\theta _s$ is the saturated water content, $\alpha$ is the air entry parameter, n is a porosity distribution measure.

The soil water matric potential levels were selected based on the precision irrigation thresholds’ distribution on the soil water retention curve to cover a wide range of field-realistic water content. The van Genuchten parameters used to create the water retention curve were optimized based on the SMP and soil water volumetric data measured throughout one growing season using optimization.

Table 2 shows the soil volumetric water content and volumetric air content for each irrigation threshold.

**Table 2:** Volumetric soil water and air content (cm$^3$/cm$^3$) for each irrigation threshold.

Irrigation thresholds	Equivalent water content (cm$^3$/cm$^3$)^a^	Equivalent air content (cm$^3$/cm$^3$)^a^
$-15$	0.35	0.13
$-30$	0.33	0.15
$-45$	0.32	0.16
$-60$	0.31	0.17

^a^Calculated with the van Guenuchten equation^47^.

### Greenhouse environmental conditions

During the incubation period of the 2018 experiment, the air temperature of the soil at 10-cm depth was measured using GS3 probes (Decagon Devices, Inc., Pullman, WA, USA) at 15 minutes intervals. It varied between 14.9 and 29.8 $^\circ$C. No significant difference in the averages air temperature of the soil were observed between $-15$ kPa, $-30$ kPa, $-45$ kPa, and $-60$ kPa treatment (ANOVA, *p*-value > 0.05), respectively, 18.7 $^\circ$C ± 3.00, 18.4 $^\circ$C ± 3.10, 18.7 $^\circ$C ± 3.00 and 18.5 $^\circ$C ± 2.80. Therefore, soil air temperature did not show any significant effect on the TBI. Soil temperature between treatments was not significantly different.

The environmental conditions in the greenhouse were measured with a complete weather station set inside the greenhouse. During the incubation period of the three experiments, air temperature in the greenhouse varied between 12.42 and 31.46 $^\circ$C with a mean of 19.74 $^\circ$C ± 3 $^\circ$C. The mean air temperature for winter 2018, winter 2019, and summer 2019 were respectively 18.67 $^\circ$C ± 2.24 $^\circ$C , 21.58 $^\circ$C ± 2.88 $^\circ$C and 19.11 $^\circ$C ± 3.02 $^\circ$C . During the three experiments’ incubation period, the relative humidity varied between 8.08 and 99.5%, with a mean of 44.81% ± 16.15%.

During the growing season of the three experiments, the cumulative water supply for the $-15$ kPa, $-30$ kPa, $-45$ kPa and $-60$ kPa precision irrigation thresholds were respectively 673.19 mm ± 16.27 mm, 449.51 mm ± 13.32 mm, 312.58 mm ± 8.66 mm, 254.05 mm ± 9.23 mm.

### Tea bag index parameters

The two tea types have significantly different decomposition properties: a slow decomposition rate characterizes the rooibos tea, and a fast decomposition rate characterizes the green tea. The decomposition rate difference between the two types allows the construction of a decomposition curve using a single measurement in time^30^. The calculation of the TBI indices is done in five steps. First, the decomposed fraction of green tea (ag) is calculated using Eq. (1).

1 $$\begin{aligned} ag = 1- \frac{m_{Tf}}{m_{Ti}} \end{aligned}$$

where $m_{Tf}$ is the final mass of green tea and $m_{Ti}$ is the tea initial mass in grams. Secondly, the S factor is calculated using Eq. (2),

2 $$\begin{aligned} S = 1-\left( \frac{ag}{Hg} \right) \end{aligned}$$

where ag is the decomposed fraction of green tea and Hg is the hydrolyzable fraction of green tea ($0.842$ g g$^{- 1}$)^30^. Thirdly, the predicted labile fraction of rooibos tea (ar) is calculated using Eq. (3),

3 $$\begin{aligned} ar = Hr \cdot (1 - S) \end{aligned}$$

where Hr is the hydrolyzable fraction of the rooibos tea ($0.552$ g g$^{- 1}$)^30^ and S is the stabilization factor. Fourthly, the remaining fraction of rooibos tea (wt) is calculated using Eq. (4).

4 $$\begin{aligned} wt=\frac{m_{Rf}}{m_{Ri}} \end{aligned}$$

where $m_{Rf}$ is the rooibos final mass and $m_{Ri}$ is the initial rooibos mass in grams. Finally, the decomposition rate (k) is calculated using Eq. (5),

5 $$\begin{aligned} k= \frac{log\left( \frac{ar}{(wt-(1-ar))} \right) }{t} \end{aligned}$$

where ar is the predicted labile fraction of rooibos tea, wt is the remaining fraction of rooibos tea, and t is the incubation time of teabags.

### Teabags preparation and incubation

In this study, 1.77 g of green tea (Lipton green tea, EAN: 87 22700 05552 5) and 1.90 g of rooibos tea (Lipton rooibos tea, EAN: 87 22700 18843 8) were weighed to four decimal places, embedded in plastic tea bags and labeled. A pair (one green tea and one rooibos tea) of teabags was buried in the soil at 8 cm deep in each experimental unit (n = 168) at an equal distance from the irrigation system the same week as the potato emergence. The teabags were buried using a hand trowel to minimize soil and roots disturbance. The labels were kept above the soil, and the teabags emplacements were marked with plastic straws. The teabags have been recovered the day of the potato harvest and kept in the fridge at 4 $^\circ$C until lab analysis. After all soil particles and roots have been removed from the bags, the tea was removed from the bag to dry 48 hours at 70 $^\circ$C. Then, the cleaned and dry tea was weighted at four decimals.

### Weight loss-on-ignition variations

Soil organic matter variations between planting and harvest were measured using the weight loss-on-ignition method^51^ using composite soil samples from each experimental unit gathered at planting and harvest at 8-cm depth using a hand shovel and stored at 4 $^\circ$C. Afterward, the samples were screened at 2 mm, and 10 g were collected and analyzed using the weight loss-on-ignition method. The soil organic matter result was then converted into SOC using the van Bemmelen factor, considered as a reasonable predictor of SOC in soil organic matter^29^.

### PLS regression and statistical analysis

In this study, we used the PLS regression, one of the two basic PLS methods^52^, to analyze the temporal effect of daily SMP (predictors) on labile SOC decomposition rate (response) determined with the TBI method. The PLS regression method simultaneously and iteratively decompose the predictors ($X$) and response ($Y$) variables into components (called latent vectors) that explain as much as possible the covariance between predictors and response(s)^53^. For each iteration, this decomposition generates three categories of vectors: $a$) a set of orthogonal factors named score matrix ($T$ for $X$ and $U$ for $Y$), b) a set of loadings (non-orthogonal) named loading matrix ($P$ for $X$ and $Q$ for $Y$), and c) the regression weights ($w$ for $X$ and $c$ for $Y$). Then, the score matrix of the predictors, a diagonal matrix with the regression weights of the response variable(s) as diagonal elements, and the loading matrix (transposed) of the response (s) variable(s) are used to predict the response(s) variable(s). The raw predictors and responses data may be transformed (e.g., logarithmically), centered, and scaled before the model development phase for each variable to have the same probability of influencing the model. Thus, the PLS regression model is given by:

6 $$\begin{aligned} X= & {} TP^{T}+E \end{aligned}$$

7 $$\begin{aligned} Y= & {} UQ^{T}+F \end{aligned}$$

8 $$\begin{aligned} U= & {} TD \end{aligned}$$

where $E$ and $F$ are matrices containing residuals for $X$ and $Y$, respectively, and $D$ is a diagonal matrix representing the scaling of the bilinear inner relationship between $T$ (the scores of $X$) and $U$ (the scores of $Y$). As shown in Eqs. 6 to 8, $D$ is determined from $U$ and $T$. The predicted $Y$ is obtained using Eq. (9):

9 $$\begin{aligned} {\widehat{Y}}=TDQ^T \end{aligned}$$

In summary, the PLS calculates the soil matric potential weights, separately of other properties (SOC decomposition rate). The data are centered, and then the covariance between the linear combination of $X$ and $Y$ is maximized through a least-squares solution. A step-by-step procedure with examples that solve these equations is presented in Krishnan et al. (2011)^52^. The PLS regression allowed the determination, by correlation, of the most determinant period of the season in the development of the decomposition rate using only the TBI measurements and daily soil water matric potential. The optimal number of latent components used in the model was determined using cross-validation protocols, and components with an importance of over 85 were chosen as critical periods. The variable importance calculation was based on weighted sums of the absolute regression coefficients using the varImp function of the caret packages^54^.

All data were analyzed using R software^55^. The PLS analysis was made using the packages caret^54^ with a cross-validation control procedure. The variance homogeneity was acknowledged using the Levene test from Car packages^56^. The difference in TBI indices under the different precision irrigation thresholds was compared using a linear mixed model with the lme4 package^57^. The differences were considered significant when *p*-values were under 0.05.
